# Molecular Insights in Uterine Leiomyosarcoma: A Systematic Review

**DOI:** 10.3390/ijms23179728

**Published:** 2022-08-27

**Authors:** Radmila Sparić, Mladen Andjić, Ivana Babović, Lazar Nejković, Milena Mitrović, Jelena Štulić, Miljan Pupovac, Andrea Tinelli

**Affiliations:** 1 Clinic for Gynecology and Obstetrics, University Clinical Centre of Serbia, Koste Todorovića 26, 11000 Belgrade, Serbia; 2 Faculty of Medicine, University of Belgrade, 11000 Belgrade, Serbia; 3 Clinic of Gynecology and Obstetrics Narodni Front, 11000 Belgrade, Serbia; 4 Department of Obstetrics and Gynecology, and CERICSAL (CEntro di Ricerca Clinico SALentino), “Verisdelli Ponti Hospital”, Via Giuseppina Delli Ponti, 73020 Scorrano, LE, Italy

**Keywords:** uterine fibroid, leiomyosarcoma, molecular, gene, differential diagnosis, omics

## Abstract

Uterine fibroids (UFs) are the most common benign tumors of female genital diseases, unlike uterine leiomyosarcoma (LMS), a rare and aggressive uterine cancer. This narrative review aims to discuss the biology and diagnosis of LMS and, at the same time, their differential diagnosis, in order to distinguish the biological and molecular origins. The authors performed a Medline and PubMed search for the years 1990–2022 using a combination of keywords on the topics to highlight the many genes and proteins involved in the pathogenesis of LMS. The mutation of these genes, in addition to the altered expression and functions of their enzymes, are potentially biomarkers of uterine LMS. Thus, the use of this molecular and protein information could favor differential diagnosis and personalized therapy based on the molecular characteristics of LMS tissue, leading to timely diagnoses and potential better outcomes for patients.

## 1. Introduction

Uterine fibroids (UFs), or leiomyoma, are the most common genital benign tumor in the female population [1]. UF affects up to 70% of women during their lifetime, and it has a great influence on health and the quality of life as well as economic costs [1,2,3,4]. Nevertheless, the pathogenesis is not yet fully understood nor is there a conservative effective therapy for UF. It has been observed that around 30% of patients with UFs show symptoms, including abnormal uterine bleeding, pelvic pain and pressure, anemia, back pain, constipation and urinary frequency that is dependent on the UFs’ localization and size [5]. It was reported that 71% of UFs diagnosed on symptomatic women used pharmacological therapy and that about 30% of patients underwent surgical or interventional radiology procedures for symptom relief [6]. 

UF management includes a wide spectrum of treatments, ranging from pharmacological therapy (including selective progesterone receptor modulators (SPRMs)) to surgical procedures, such as myomectomy by hysteroscopy, laparotomy or laparoscopy, hysterectomy, uterine artery embolization and radiological interventions [7]. The management strategy depends on the patient’s age and wish for childbearing and, on the other hand, on the number, size and location of UFs [7]. The laparoscopic myomectomy is one of the best surgical options for UF management in women wishing to preserve their fertility. 

Compared to laparotomy, laparoscopic myomectomy leads to a shorter hospital stay, less postoperative pain, faster postoperative recovery, positive reproductive outcomes, less morbidity and reduced adhesion formation [8,9,10]. On the other hand, laparoscopic myomectomy can show greater blood loss, longer operative times, increased risk of recurrence, as well as increased risk of uterine rupture in future pregnancies and a potential dissemination of myoma and/or undiagnosed LMS cells by morcellation [9]. Moreover, one of the greatest problems in gynecological laparoscopic surgery is the removal of large masses, such as huge fibroids, from the abdomen or pelvis. 

One common solution for fibroids removal is the morcellation, which causes the fragmentation into small pieces of large masses, which are then retrieved from the abdominal cavity [11]. However, the Food and Drug Administration (FDA) warned about the use of morcellation in 2014 because of the risk of undiagnosed uterine leiomyosarcoma (LMS) in women undergoing morcellation during laparoscopic hysterectomies and myomectomies [12]. Uterine LMS represents a rare malignant and aggressive uterine cancer with an unfavorable prognosis and the highest prevalence in pre- and peri-menopause women. 

**Figure 1:**
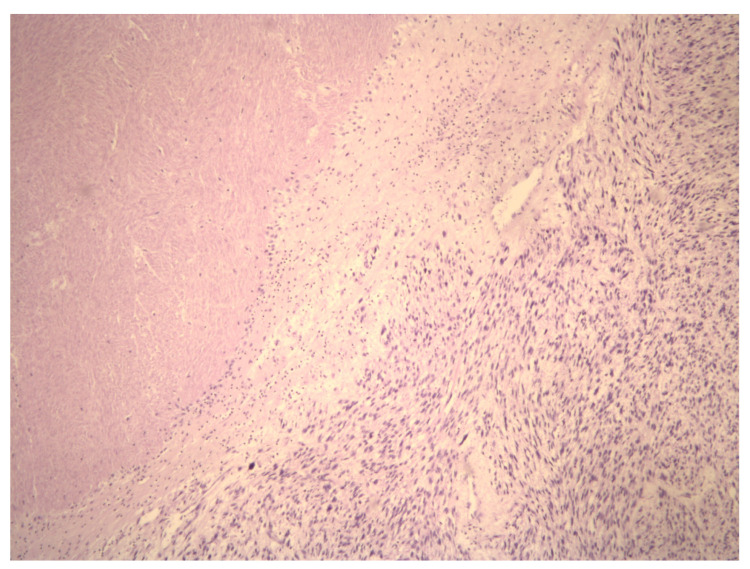
Aview of the pathohistological preparation of LMS cells (magnification at 5×) showing the coagulative necrosis of LMS cells. This highlights the demarcation of area of viable and necrotic LMS cells.

**Figure 2:**
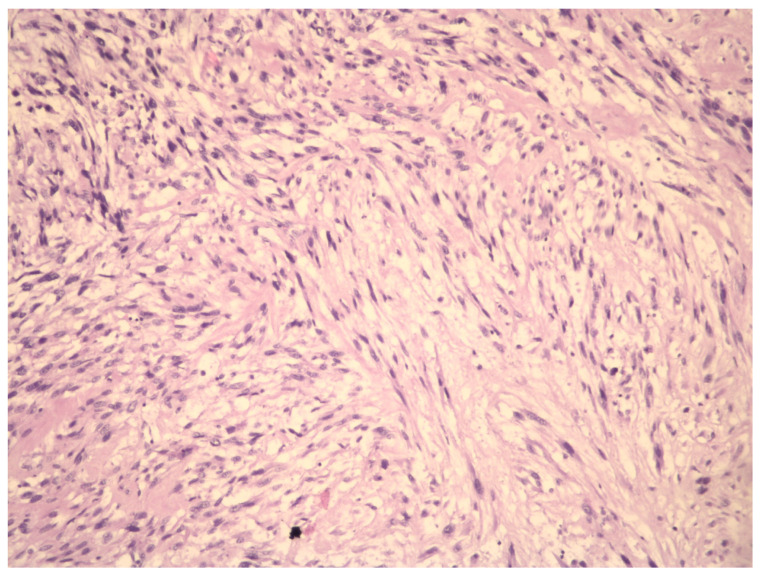
Aview of the pathohistological preparation of LMS cells (magnification at 10×). The cytologic atypic LMS cells have large, irregularly shaped and pleiomorphic nuclei with scarce chromatin.

Generally, a uterine LMS is accidentally diagnosed during an anatomopathological analysis of removed tissue, such as uteri or UFs for benign uterine diseases. In this last case, unfortunately, if morcellation isused, there is a risk of dissemination of uterine LMS cells and a worsening prognosis for these patients [13]. However, the current molecular techniques provide either scientific evidence on uterine LMS pathogenesis or some possible solutions for the LMS preoperative diagnosis, based on the differential diagnosis between UF and uterine LMS. This narrative review aims to discuss LMS biology and diagnosis (Figure 1 and Figure 2) and molecular variations that could be possible biomarkers of uterine LMS.

## 2. Methods

The authors searched the available data on the molecular basis of UF pathogenesis, diagnosis and prognosis of UF and LMS. The authors conducted a MEDLINE, Scopus and PubMed search, for the years 1990–2022, using a combination of keywords, such as “uterinefibroid, “myoma,”“fibromyoma”, “leiomyoma”, “myomectomy,”, “molecular”, “genetic”, “prognosis”, “leiomyosarcoma”, “management”, “treatment” and “differential diagnosis. Randomized controlled trials (RCTs) were used when available; otherwise, the literature that was the most relevant to the topic was used based on the authors’ evaluation. 

Peer-reviewed articles concerning uterine fibroids, myomas and leiomyomas were included in this paper. Additional articles were identified from the references of relevant papers. The terms “uterine fibroids”, “myomas”, “fibromyomas” and “leiomyomas” can also be found in the literature describing UFs. In the manuscript, we use the terms “fibroid” and “myoma” in equal measure. 

## 3. Results and Discussion

**Figure 3:**
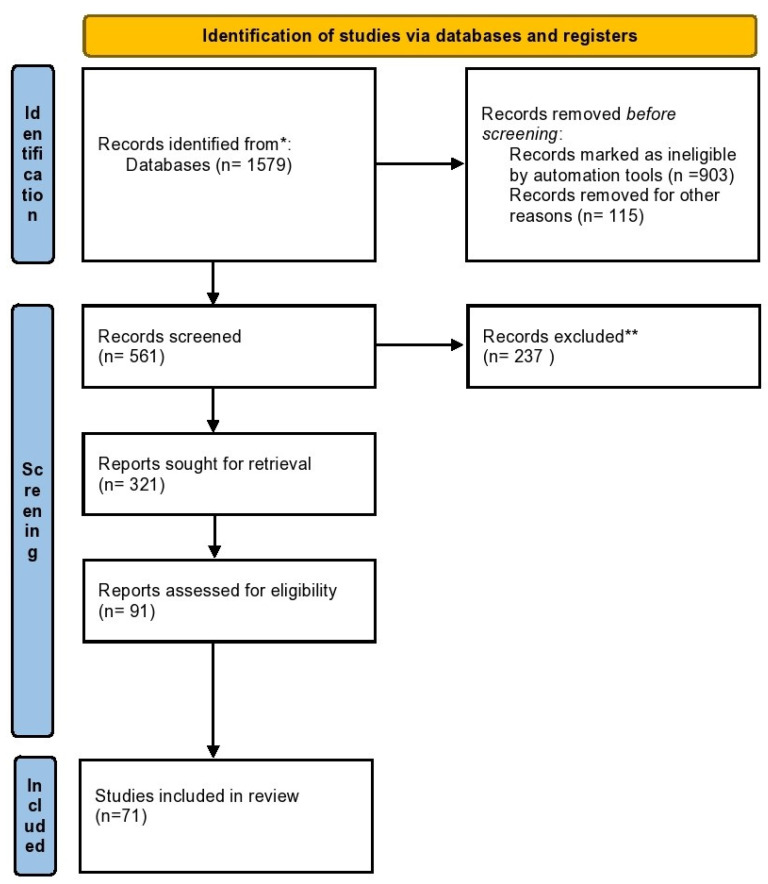
The flow chart illustrating the study methodology. Legend: * Scopus, Medline and Pubmed bases; **references that are not relevant to the topic.

The research methodology is included in a flow chart (Figure 3). The results of the research are divided into different paragraphs with which we illustrate what has been reported in the scientific literature.

### 3.1. Genetic Changes Linked to Leiomyosarcomas Pathogenesis, Diagnosis and Prognosis

**Figure 4:**
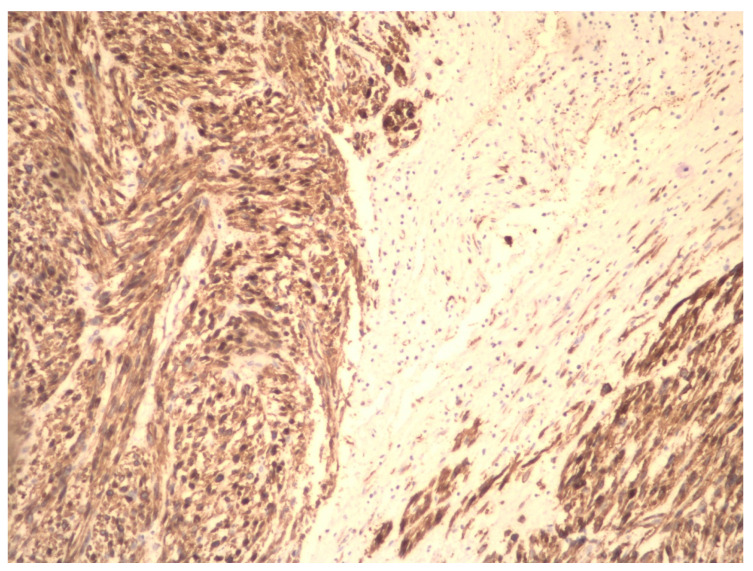
Immunohistochemical preparation of LMS cells (magnification at 10×). The tumor is heterogeneous with an area of necrosis. The tumor cells express smooth muscle actin.

Although early-stage uterine LMS has an acceptable prognosis, generally, uterine LMS is diagnosed in advanced stages and occasionally, after anatomic–pathological examination and/or immunohistochemical analysis of a fibrotic uterus or UFs (Figure 4).

LMS prognosis is generally poor but may even worsen after vaginal and abdominal morcellation during minimally invasive surgical procedures for common benign gynecological diseases [13]. The molecular diagnosis in the era of omics could provide new knowledge that may prove useful for early and differential diagnosis and possible LMS therapy. Recent investigations showed that certain gene mutation and chromosomal abnormalities could be potential biomarkers of LMS. Zhang et al. [14] performed bioinformatics analysis to identify the key genes and pathways that have roles in the uterine LMS development. The authors reported 21 upregulated genes and 74 downregulated genes with possible roles in uterine LMS development. 

The selected upregulated genes have a role in “DNA metabolic process”, “nucleobase-containing compound biosynthetic process” and “cellular macromolecule biosynthetic process”, while the downregulated genes were linked to the “cellular response to chemical stimulus”, “movement of a cell or subcellular component” and “response to inorganic substances”. The authors reported that matrix metalloproteinase 9), apolipoprotein E (apoE), cyclin E1 and syndecan 1 were upregulated genes in uterine LMS. Chen et al. [15] investigated the role of the shank-associated RH domain-interacting protein (SHARPIN) gene in uterine LMS appearance. 

They reported four genes—solute carrier family 39 (zinc transporter) member 7 (SLC39A7), G-protein-coupled receptor 19 (GPR19), zinc finger protein 17 (ZNF717) and tumor protein 53 (TP53)—that could be driver mutations. The authors observed gain in regions 1q21-23, 19p13, 17q21 and 17q25 in the chromosomes, as well as loss in regions 2q35, 2q37, 1p36, 10q26, 6p22, 8q24, 11p15, 11q12 and 9p21 in the chromosomes. TheSHARPIN gene was amplified in some patients with uterine LMS. 

These findings suggest SHARPIN gene as a new diagnostic and therapeutic marker of uterine LMS. Hensley et al. [16] performed a prospective molecular characterization of LMS. They correlated the genomic landscape with survival and therapeutic targetability. 

According to their results, common genetic alterations in uterine LMSs were functional mutations in TP53 (56%), retinoblastoma protein 1 (RB1) (51%) and alpha-thalassemia/mental retardation X-linked (ATRX) (31%). In addition, the alteration of phosphatase and tensinhomologue (PTEN) gene was more frequent in LMS metastases than in samples of primary LMS. The high-grade tumors had more common duplication of the whole-genome in comparison with low-grade tumors. Makinenet et al. [17] performed an exosome sequencing in 19 LMS samples to investigate somatic gene variation in uterine LMS. The authors reported mutated genes, including TP53 (6/19, 33%), mediator complex subunit 12 (MED12) (4/19; 21%) and ATRX (5/19; 26%).

The authors reported that both TP53 and MED12 genetic alterations were linked to uterine LMS. Moreover, they reported that a reduction in the expression of ATRX was linked with alternative lengthening of telomeres (ALT). The ALT phenotype was commonly seen in tumor candidates, and ATR inhibitors could be a new potential therapeutic approach for uterine LMS. Astolfi et al. [18], in a study including 216 patients, observed that the majority of patients with uterine LMS carried at least one mutation in either the TP53, RB1, ATRX or PTEN genes and that mutation in the TP53 gene was most common, at 61% of the patients, and RB1 was found in 48% of the patients. 

The authors showed that the PTEN gene mutation was more common in LMS metastases than in primary LMS. Interestingly, TP53 and RB1 mutations occurred together, and, in the TP53-mutant subgroup, the RB1 mutation had a favorable prognostic significance. Lee et al. [19] analyzed genomic alterations in canonical cancer-related genes in LMS, including small insertions/deletions, single nucleotide variants and copy number alterations. They showed that the most common alterations were observed in the TP53 (36%), ATM and ATRX (16%) and EGFR and RB1 (12%) genes.

The authors observed that about 85% of cases of LMS had alterations in the gene copy number, and that chromosomes 10 and 13, including PTEN and RB1, had the most common losses in gene copy number, while chromosomes 7 and 17 had the most common gains in gene copy number. The observed data suggest that deletions in cancer-related genes are common in LMS. The observed gene mutations imply that defects in chromosomal maintenance and DNA repair have a central role in the development of LMSs and, at the same time, activating mutations—which are common in other cancer types - are rare in LMSs.

Similarly to other genetics investigations, Choi et al. [20] observed recurrent somatic mutations in the TP53, MED12 and PTEN genes. The TP53, ATRX, PTEN and MEN1 genes have numerous somatic mutations. They reported eight copy-number gains, including 5p15.33 (TERT), 8q24.21 (C-MYC) and 17p11.2 (MYOCD andMAP2K4) amplifications and 29 copy-number losses. They observed fusions in tumor suppressor andoncogenes. The RB1, TP53 and ATRX/DAXX had the most disrupting fusions. The fusion (ACTG2-ALK) was potentially targetable. 

In addition, it has been noted that 76% of the LMS samples have chromothripsis and/orchromoplexy. Microsatellite instability (MSI) and homologous-recombination DNA-repair deficiency were identified in 2% and 25% of LMS samples, respectively. Cuppens et al. [21] reported that genomic alterations affecting TP53,RB1,PTEN,MED12,tyrosine 3-monooxygenase/tryptophan 5 monooxygenase activation protein epsilon tyrosine(YWHAE) and vasoactive intestinal peptide receptor 2 (VIPR2) were present in the majority of uterine LMSs. 

The known oncogenes (cyclin E1 and tryptophan 2,3 dioxygenase (TDO2)) were over expressed in uterine LMS samples, and the tumor suppressor genes PTEN and PR/Set domain 16 (PRDM16) were influenced by reduced expression as well as deletions. The authors showed that the gene with most frequent mutations in the samples in their study wasVIPR2 (96%). They reported also that VIPR2 deletion was associated with troublesome survival in LMS patients. When comparing VIPR2 protein expression in uterine LMSs with VIPR2 expression in normal myometrium, they observed lower expression of VIPR2 protein in LMS.

Raish et al. [22] analyzed changes in the DNA copy number in 15 uterine LMS cases. They reported that the percentages of average losses and gains were 16.6% and 8.4%, respectively. The chromosomal regions of 1q23.3, 7p14.2, 7q34, 7q35, 7q36.3, 13q34 and 16p13.3had the high levels of gains. Homozygous loss was observed in chromosomal regions 2q21.1, 2q22.1, 2p23.2, 12q23.3, 4q21.22, 4q34.3, 11q24.2, 12q23.3, 13q13.1, 13q21.33 and 14q24.3. 

In uterine LMS, regions with recurrent gain were 1p36.33, 1p36.32, 5q35.3, 7q36.3 and 8q24.3, and recurrent regions of loss were 1p31.1-p31.3, 1p32.1-p32.3, 2p12, 2p13.3, 2p14, 2p16.2-p16.3, 2q12.1-q12.3, 2q21.1-q21.2, 2q22.2-q22.3, 2q34, 2q36.1-q36.3, 5q21.3, 5q23.3, 5q31.1, 6p11.2, 6p12.1, 10q11.23, 10q21.2-q21.3, 10q23.2, 10q23.31, 10q25.1-q25.2, 10q25.3, 10q26.13, 10q26.2-q26.3, 11p11.2, 11p11.12, 11p12, 11p13, 11p15.4, 11q23.1-q23.2, 11q23.3, 13q14.12, 13q14.13-13q14.2, 13q14.2, 13q14.2, 13q14.3, 13q21.33, 13q22.1-q22.3, 14q24.2, 14q24.3, 14q31.1, 14q32.33, 15q11.2-q13, 15q14, 16q22.3, 16q23.1, 16q23.2, 16q24.1, 20p12.1 and 21q22.3. 

Davidson et al. [23] compared the gene expression in primary uterine LMS and LMS metastases. They obtained that the genes OSTN, NLGN4X, NLGN1, SLITRK4, MASP1, XRN2, ASS1, RORB, HRASLS and TSPAN7 were overexpressed in primary LMS, while the TNNT1, FOLR3, TDO2, CRYM, GJA1, TSPAN10, THBS1, SGK1, SHMT1, EGR2 and AGT genes were overexpressed in LMS metastases.

#### Epigenetic, Metabolomic and Proteomic Changes in Pathogenesis of Uterine Leiomyosarcoma

There are not only genetic alterations observed in leiomyosarcoma pathogenesis. Recent research suggested changes in the epigenetic milieu in the pathogenesis of leiomyosarcoma. Modifications in the higher methylation level of Krupel-like factor 4 (KLF4) and (dendritic cell lectin 1) DLEC-1 gene were observed in uterine LMS, compared to the normal myometrium [24]. The micro RNAs, such as small non-coding RNAs, have a biological role in the regulation of gene expression at the post-transcriptional level. A significant under-expression of two micro RNAs (miR-1-3p andmiR-202-3p) and over-expression of miR-7-5p were detected in uterine LMS. 

The altered expression of these mRNAs could lead to the disruptions in their micro RNA-target, with the possibility of causing the development of uterine LMS [25].The micro RNAs expression profile of uterine LMS also demonstrated biological similarity with other organs. The micro RNA expression profile of uterine LMS demonstrated similarity to bone-marrow-derived human mesenchymal stem cells and similarity to the fool smooth muscle cell differentiation micro RNA profile. These findings suggest that divergent pathways are involved in the pathogenesis and transformation of uterine LMS and UFs [26].

### 3.2. Molecular Basis of Differentiation Uterine Leiomyosarcoma vs. Uterine Leiomyoma

#### 3.2.1. Genetic and Gene Expression Differences between Leiomyosarcoma and Uterine Leiomyoma

Of great importance in the diagnosis of uterine LMS is the difference from uterine leiomyoma, reported in some investigations as the molecular differences between LMS and UF. Machado-Lopez et al. [27] conducted differential exome and transcriptome-wide research in histologically confirmed leiomyomas and LMSs and aimed to investigate the differences between and within these two pathologies. They reported significantly higher copy number variants and tumor mutation somatic single-nucleotide variants for the LMSs vs. the leiomyomas. 

Transcriptomic analysis within the same study observed 489 differentially expressed genes between LMS and UFs. Similar results were obtained by Mas et al. [28]. The authors observed deletions in 20 genes in LMSs, compared with only six observed gene losses in UFs. On the other hand, duplications of genes were identified in 19 genes in LMSs but in only three genes in UFs. 

The 105 genes in LMS samples were affected by insertions/deletions and single-nucleotide variants; at the same time, only 82 genes in UFs had single-nucleotide variants and insertions/deletions. The study identified the differential transcriptome profile for 11 of the 55 genes analyzed in LMSs.

Bertch et al. [29] reported that MED12 mutations and high-mobility-group AT-hook 2(HMGA2) overexpression are genetic events in UF leiomyomas and that they may have different roles in the tumorigenesis of UFs. Sahly et al. [30] analyzed the global gene expression profiles of UF and LMS myometrium tissues using human genome microarrays. There identified 249, 1037 and 716 significantly expressed genes, respectively, when comparing the expression signals across the normal myometrium vs. UFs, normal myometrium vs. LMS and UF vs. LMS groups. 

The authors observed multiple alterations of numerous key pathways in genes of the extracellular matrix, collagen, cell contact inhibition and cytokine receptors regarding the transformation of normal myometrial cells to benign leiomyomas. When the authors compared the genetic alterations between UFs and uterine LMSs, they found affected cell-cycle- and cell-division-related Rho GTPases and PI3K signaling pathways triggering uncontrolled growth and metastasis of tumors. The authors found differences in thedistribution of hubs (JUN, VCAN, TOP2A and COL1A1) and eight bottleneck genes (PIK3R1, MYH11, KDR, ESR1, WT1, CCND1, EZH2 and CDKN2A) among UFs and LMSs.

Zhang et al. [31] investigated the differences in 17 LMS relevant biomarkers in four tumorigenic pathways, including steroid hormone receptors (AKT pathway markers, estrogen receptor-ER and progesterone receptor-PR), cell cycle/tumor suppressor genes and associated oncogenes. The 119 patients diagnosed for uterine smooth muscle tumors were divided into the following groups: LMS, smooth muscle tumor of uncertain malignant potential, atypical myomas/myoma with bizarre nuclei and cellular myoma and 60 myometrial controls. 

The ER and PR were expressed lower in the LMS samples, in comparison to other uterine smooth muscle tumors. Although there was a difference in the expression of cell cycle genes in different types of tumors, the authors observed a significant overlap. The Ki-67 index was greater than 33 in 75% of cases of LMS, while only 5% of other types of uterine smooth muscle tumors samples had aKi-67 index greater than 33. The cell proliferative indices (Ki-67) and sex steroid hormone receptors expression could differentiate LMS from other types of uterine smooth muscle tumors.

Baiocchiet al. [32] analyzed the expression of TOP2A in 37patients with LMS, in 12 patients with myoma variants, in four patients with smooth muscle tumors of uncertain malignant potential and in 23 patients with uterine leiomyomas. They observed that TOP2A was highly expressed in LMS but not in non-malignant diseases. Adams et al. [33] analyzed gene expression profiles in LMS samples, in myoma and in normal myometrial samples to identify a biomarker for discriminating between LMS and myomas. They showed that CHI3L1, MELK, PRC1, TOP2A and TPX2 genes were overexpressed in LMSs, while HPGD and TES genes were overexpressed in UFs. 

These genes’ expression, identified as a potential biomarker for LMS and myoma uteri, could provide a potential prognostic information and be novel pharmacological targets in the LMS treatment. Banas et al. [34] compared the DNA fragmentation factors 40 and 45 (DFF40 and DFF45 and Bcl-2 (B-cell lymphoma 2) expression in LMS, UFs and normal myometrial tissue. They correlated these gene modifications with the disease-free and overall survival, reporting that DFF40, DFF45 and Bcl-2 were significantly under expressed in uterine LMS compared with myoma and normal myometrium. The disease-free survival and overall survival of patients with LMS was negatively influenced by the lack of expression of DFF40 and Bcl-2.

#### 3.2.2. Micro RNAs as Potential Biomarkers for Differential Diagnosis between Uterine Leiomyosarcoma and Uterine Leiomyoma

Recent investigations showed that genome alteration and gene expression products are biomarkers for LMS and UF differential diagnosis; however, micro RNAs could also be included with these. Yokoi et al. [35] considered circulating micro RNAs as diagnostic biomarkers for the differentiation of LMSs from UFs with an emphasis on circulating micro RNAs. They analyzed the expression levels of the micro RNAs. The candidate micro RNAs were selected based on their diagnostic performance in discriminating LMSs from UFs, and then the authors constructed a diagnostic model. 

They constructed a model that included two micro RNAs (miR-1246 and miR-191-5p) with an area under the receiver operating characteristic curve (AUC) for diagnosing an LMS of 0.97. Additionally, the authors detected seven serum micro RNAs for preoperative US screening. Hu et al. [36] compared the expression of STMN1 and MKI67 micro RNA in uterine LMS, UFs and uterine cellular leiomyoma (UCL) and uterine normal smooth muscle tissue (UNSM) (30 UNSM, 30 UF, 24 UCL and 18 uterine LMS). They observed significant upregulation in the STMN1and MKI67micro RNA and protein expression levels in uterine LMS comparison with the other three groups. 

De Almeida et al. [37] evaluated the expression profile of the lethal-7(let-7) family—an important micro-RNA group of tumor suppressors—and their prognostic value in uterine LMS. The micro RNAs expression profile was obtained from 34 LMSs and 13 myometrium samples. The authors obtained that all the let-7family members’ expression was downregulated in LMS patients, observing that Let-7e expression was linked to worse overall survival, while let-7b and let-7d expression were associated with worse disease-free survival.

The potential molecular biomarkers for differential diagnosis between uterine LMS and UFs are presented in Table 1.

There are numerous gene as well as their protein products involved in LMS pathogenesis. The mutation of these genes and altered expression and function of their expressed products are potentially biomarkers of LMSs. The PTEN gene is a tumor-suppressor gene whose function depends on the PI6/K/AKT/mTOR growth-promoting signaling cascade. PTEN dysfunction and the consequent dysregulation of this and other pathways are involved in many cancers [38]. As the literatureshows, transcriptional dysregulation in the p53 signaling pathway, PI3K/AKT/mTOR pathway and the Wnt signaling pathway has also been implicated in the pathogenesis of many cancers. 

The researchers observed significantly reduced expression of PTEN, the negative regulator of PI3K/AKT/mTOR signaling pathway, in more than half of uterine LMS [15]. PTEN also has an influence on the DNA damage response tumor immune micro-environment [39]. There is evidence suggesting PTEN as an anti-tumor immunity promoter. It was revealed to increase the levels of pro-oncogenic inflammatory cytokines (such asCCL2) immunosuppressive cells (such as MDSCs and Tregs) and reduce the levels of NK cells, helper T cells and cytotoxic T cells in patients that had tumors with PTEN deficiency [40].

MMP-9isa zinc-dependent proteolytic metalloenzyme, belonging to the family ofmatrix metalloproteinases that are involved in the degradation of components of the extracellular matrix with roles in physiological and pathological processes [41]. MMP-9 maybe related to some cancers, including neoplastic invasion, metastasis and neoangiogenesis [42]. The upregulated gene of MMP-9 has an influence on tumor progression due to its ability to degrade the components of extracellular matrix. ERK activation leads to the upregulation of MMP-9 expression and subsequent MMP-9-mediated shedding of SDC1 [43]. 

MMP-9 has many different biological targets (cytokines, growth factors, chemokines, cytokine/growth factor receptors and the extracellular matrix) and modulates the cell growth, invasion, migration, angiogenesis and inflammation signaling pathways. Gelatinases’ active site or the hemopexin-like C-terminal domain is a mediator with cell-surface-integral membrane proteins (CD44, αVβ/αβ1/αβ2 integrins and the Ku protein) [44].It is also known that SDF-1/CXCL12 accelerates the shedding of SDC1/MMP-9in human primary macrophages and HeLa cells and that theMMP-9-miR-494-SDC1 regulatory loop is linked to irradiation-induced angiogenesis in medulloblastoma cells. 

miR-494 suppression by MMP-9 enhances angiogenesis and SDC1 shedding [45,46]. It has also been reported that MMP-9 has a potential role in other tumors, such as breast cancer, where fibroblasts promote angiogenesis and then enhance tumor growth by the upregulation of MMP-9 via the MAPK-AP1 signaling axis, which is co-stimulated by TGF-β, TNF-α and IL-1β [47]. The altered expression of SDC1 gene alters the encoding of the type-1 transmembrane heparan sulfate proteoglycan (HSPG), which is involved in cell growth and migration, cell–cell and cell–matrix interactions as well as adhesion-dependent signaling pathways and neovascularization [48]. 

Syndecan—as one of the extracellular matrix components—binds growth factors and cytokines and modulates the tumor micro-environment [49]. On the other hand, syndecan also has an essential role in tumorigenesis, induced by the Wnt-1 signaling pathway [50]. Decreased SDC1 expression leads to the increase of activation of β1-integrins, focal adhesion kinase and Wnt signaling, which are all linked to aggressive cancer growth [51]. 

In interactions with laminin 332, syndecan was shown to promote tumor invasion via the PI3K and RAC1 signaling pathways [52]. It was found that SDC1 internalizes Apolipoprotein E, avery low-density lipoprotein (ApoE-VLDL) independently of the low-density lipoprotein-receptor-related protein (LRP) pathway. The altered expression of the ApoE gene, obtained in LMS analysis, means that ApoE may be related to oncogenesis. It has been reported that the serum levels of ApoE are elevated in patients within breast cancer and non-small-cell lung cancer [53,54].

The protein cyclin E1, also down-regulated in LMSs, is a regulator of the G1/S transition and suppressor of the RB protein, by activating cyclin-dependent kinases (CDK) and leading to uncontrolled proliferation. During physiological conditions, Cyclin E1associated to CDK2 promotes G1/S transition. This protein complex regulates cell-cycle progression and DNA replication through the phosphorylation of specific substrates. In oncogenic conditions, the Cyclin E/CDK2 complex affects normal DNA replication, causing DNA replication stress, leading to cellular instability—a precursor of cancer development [55].

It has also been observed that the SHARPIN gene was over expressed in LMS. This molecule has an essential role in normal tissue development as well as pathological process inflammation, homeostasis and carcinogenesis. Similar as in LMS, the SHARPIN gene is upregulated in many human cancers, including breast cancers, hepatocellular carcinoma and melanomas. The SHARPIN gene induces cancer cell survival, growth, invasion and metastasis [56,57,58], and its depletion leads to increased p53 expression and decreased cancer cell proliferation. The SHARPIN gene either facilitates p53 degradation and poly-ubiquitination in an MDM2-dependent manner [59], or it leads to the cancer cell migration and invasion by regulating Ras-associated protein-1(Rap1) and its downstream signaling pathways, including JNK/c-Jun andp38 [60]. 

The SHARPIN gene interferes with Wnt/β-catenin signaling, competing with the E3 ubiquitin ligase β-Trcp1 for binding with β-catenin and leads to a decrease in β-catenin ubiquitination levels. Elevated expression of the SHARPIN gene in cancer tissues is associated with malignant potential and correlates with the expression levels of β-catenin [61]. However, considering that SHARPIN gene amplification is associated with a decrease in the overall survival and progression-free survival, further studies should determine whether SHARPIN gene can be utilized as a potential molecular marker in LMS diagnosis and therapeutic prognosis.

Three somatic mutated genes (SLC39A7, GPR19 andZNF717), identified by molecular analysis in LMSs, have been found to be involved in carcinogenesis. SLC39A7 modulates many signaling pathways, such as iMAPK, mTOR and PI3K-AKT, which are all involved in the proliferation and survival of cells [62]. The SLC39A7gene is also overactivated in other cancers of the female genital tract [63,64]. The other two somatic genes, GPR19 and ZN717, are also frequently mutated and overexpressed in other cancers [65,66]. The ATRX gene encodes a transcriptome regulator, which is a member of the SWI/SNF family involved in the chromatin remodeling proteins. 

The loss of ATRX expression is associated with altered telomeres and causes ATRX cooperate with DAXX, and DAXX mutations are associated with ALT [67]. The ATRX protein is recognized as the central caretaker of the human genome involved in the suppression of cancer development [68]. This protein is included in chromatin remodeling; however, the ATRX gene is mutated in many cancers. The mutations in the ATRX gene are linked to changes in transcription and alterations in the signaling pathways in cancer cells, such as the downregulation of the cadherin family of proteins and the upregulation of the TGF-β pathway [69].

MED12 regulates global transcription in eukaryotic cells. MED12 mutations are common in LMS. It is possible that MED12 mutations are a consequence of mutations in the UF precursors of LMS [70]. Interestingly, when comparing uterine myometrial cells with MED12 mutations and myometrial cells with a non-mutated MED12 gene, myometrial cells with MED12 somatic mutations have inhibition of autophagy. The myometrial cells with MED12 mutations show dysregulation in the oncogenic Wnt4/β-catenin pathway and its downstream mTOR signaling pathway. These findings implicate that cell proliferation and LMS development is a consequence of MED12 mutation and alterations in oncogenic Wnt4/β-catenin and its downstream mTOR signaling [71].

## 4. Conclusions

Even though there is not yet a non-invasive technique to diagnose uterine LMS, the development and the utilization of molecular techniques has had a promising influence on possible preoperative diagnoses as well as on the treatment and prognosis of uterine LMS. There are some molecular candidates for differential diagnosis between uterine LMS and leiomyoma, such as MED12, HMGA2, TOP2A, CHI3L1, MELK, PRC1, TOP2A, TPX2 DFF40, DFF45, the Bcl-2 gene, miRNAs (miR-1246 and miR-191-5p), STMN1, MKI67micro RNA and the let-7 family micro RNAs.

The results obtained from previous studies are not yet clinically applicable; however, they can be the starting point for further investigation regarding LMS pathogenesis and diagnosis. Large multicentric studies with large numbers of patients and novel molecular diagnostic techniques should be promoted. Differential diagnosis and personalized therapy based on the molecular characteristics of LMS tissue could lead to timely diagnoses and better outcomes for patients.

## Figures and Tables

**Table 1:** Potential molecular biomarkers for differential diagnosis between uterine LMSand UFs.

Molecular Biomarker	Reference
MED12 gene mutations in leiomyosarcoma	Bertch et al. [29]
Progesterone and estrogen receptor under-expression in uterine leiomyosarcoma	Zhang et al. [31]
TOP2A gene overexpression in leiomyosarcoma	Baiocchi et al. [32]
CHI3L1, MELK, PRC1, TOP2A andTPX2 gene overexpression In uterine leiomyosarcoma	Adams et al. [33]
DNA fragmentation factors 40 and 45 (DFF40 and DFF45 and Bcl-2 (B-cell lymphoma 2) under-expression in uterine leiomyosarcoma	Banas et al. [34]
miRNAs (miR-1246 and miR-191-5p) in leiomyosarcoma	Yokoi et al. [35]
STMN1 and MKI67 micro RNA are overexpressed in uterine LMS	Hu et al. [36]
let-7family micro RNA is downregulated in uterine leiomyosarcoma	De Almeida et al. [37]

## Data Availability

Data sharing not applicable.
